# CDK4/6 inhibitor combined with endocrine therapy: a real-world evaluation of efficacy and safety in HR+/HER2- advanced breast cancer

**DOI:** 10.1186/s12957-026-04486-1

**Published:** 2026-07-09

**Authors:** Rui Wang, Yuying Wang, Yaling Zeng, Xihui Tu, Di Lu, Purong Zhang

**Affiliations:** 1 Department of Thyroid and Breast Surgery, Mianyang 404 Hospital, Mianyang, Sichuan 621000 China; 2 College of Clinical Medicine, North Sichuan Medical College, Nanchong, Sichuan 637000 China; 3 Department of Health Management, Qionglai Medical Center Hospital, Qionglai, Sichuan China; 4 School of Medicine, University of Electronic Science and Technology of China, Chengdu, China; 5 Department of Breast Surgery, Affiliated Cancer Hospital of University of Electronic Science and Technology of China, Chengdu, 610000 China

**Keywords:** CDK4/6 inhibitor, Endocrine therapy, Advanced breast cancer, Hormone receptor positive, Human epidermal growth factor receptor 2 negative

## Abstract

**Background:**

The combination of cyclin-dependent kinase 4/6 inhibitor (CDK4/6i) with endocrine therapy (ET) constitutes the standard treatment for Hormone receptor-positive (HR+)/Human epidermal growth factor receptor 2-negative (HER2-) advanced breast cancer (ABC). It is essential to evaluate the efficacy and safety profiles of CDK4/6i in Chinese patients with HR+/HER2- ABC, in order to provide a theoretical foundation for developing precision-based therapeutic strategies.

**Methods:**

Clinical data of patients with ABC treated with CDK4/6i combined with ET between April 2021 and April 2024 at the Department of Breast Oncology, Sichuan Cancer Hospital, were retrospectively collected. Clinicopathological characteristics and prognostic outcomes were analyzed to evaluate the efficacy and safety of CDK4/6i-based regimens, and then we explored factors associated with progression-free survival (PFS). The secondary endpoints incorporate overall survival (OS), objective response rate (ORR) and clinical benefit rate (CBR).

**Results:**

A total of 124 patients were enrolled, with a median follow-up period of 23.0 months. The median PFS (mPFS) was 20.0 months, while the median OS (mOS) was not reached. Patients without measurable target lesions were excluded, and a total of 90 patients were included for further analysis. The ORR was 46.7% (42/90), and the CBR was 53.3% (48/90). Cox multivariate regression analysis identified Ki-67<20%, Age-adjusted Charlson Comorbidity Index (aCCI)<10, line of CDK4/6i ≤ 2, and sensitivity or acquired resistance to ET as independent predictors of PFS. Leukopenia (83.1%) was the most common adverse event (AE), with 75.8% of these cases classified as grade 1–2.

**Conclusion:**

This real-world (RWD) study in China has further confirmed the efficacy and safety in HR+/HER2- ABC. These findings provide further evidence for clinically identifying optimal patient population likely to derive superior benefits from CDK4/6i combination regimens.

## Introduction

Breast cancer (BC) remains the leading malignancy threatening women’s health globally. HR+/HER2- BC accounts for approximately 70% of all subtypes [1], while ET has long been the cornerstone of treatment. However, due to tumor heterogeneity-associated signaling pathway dysregulation and downregulated receptor expression, the efficacy of ET in ABC patients remains suboptimal. The FIRST study [2] established the foundation of fulvestrant in ABC treatment, while subsequent trials such as FALCON [3] and CONFIRM [4] demonstrated the superiority of fulvestrant monotherapy over aromatase inhibitors (AI) in first- and second-line settings for ABC. Nevertheless, clinical benefits of fulvestrant remain limited, particularly in patients with visceral metastases. The emergence of CDK4/6is has revolutionized the treatment paradigm. CDK4/6 regulate the transition from the G1 to S phase of the cell cycle. The PALOMA [5], MONARCH [6] and MONALEESA [7] series, further confirmed the comprehensive benefits of CDK4/6i-based therapy in HR+/HER2- ABC.

In China, four CDK4/6is—Palbociclib, abemaciclib, ribociclib and dalpiciclib—are clinically available. Although pivotal Randomized Controlled Trials (RCTs) (e.g., PALOMA, MONARCH, MONALEESA, DAWNA) have validated the efficacy and safety of these CDK4/6is, RCT outcomes may diverge from RWD clinical practice due to stringent eligibility criteria, standardized interventions, and lack of head-to-head comparisons. Moreover, delayed clinical access and economic constraints in China have resulted in limited RWD evidence on CDK4/6i use, underscoring the need for robust RWD data to optimize patient selection and individualized treatment strategies.

Based on the performance of CDK4/6is in clinical trials, combined with Chinese socioeconomic factors and various clinicopathological factors, it becomes even more important to seek more individualized treatment plans for patients. Above all, this retrospective study analyzed the efficacy and safety of CDK4/6i combined with ET in patients with HR+/HER2- ABC treated at Sichuan Cancer Hospital. Additionally, we explored clinicopathological factors associated with treatment outcomes to provide evidence for precision therapeutic decision-making.

## Materials and methods

### Study design

This retrospective study enrolled patients who received CDK4/6i combined with ET at Sichuan Cancer Hospital between April 2021 and April 2024. Inclusion and exclusion criteria were as follows. Inclusion criteria: 1) Pathologically confirmed HR+/HER2- locally advanced or metastatic breast cancer (MBC), including patients with newly diagnosed advanced disease or recurrent/metastatic disease;2) Eligible for CDK4/6i therapy without contraindications, and received CDK4/6i combined with ET during the study period;3) Patients with or without prior ET were included;4) Presence of at least one measurable lesion or osteolytic lesion;

5) Complete clinicopathological data available. Exclusion criteria: 1) Bilateral breast cancer;2) Inflammatory breast cancer;3) Loss to follow-up during the study period. Of the 143 patients initially screened based on treatment sequence and eligibility criteria, 124 met the inclusion criteria. All patients were followed for at least 6 months, with the last follow-up conducted in October 2024. Clinicopathological data were collected via electronic medical records, outpatient/inpatient reviews, and telephone follow-ups. This study was approved by the Ethics Committee of Sichuan Cancer Hospital (Approval No. SCCHEC-02-2025-086).

### Staging and pathological assessment criteria

Staging criteria: Clinical staging was performed according to the TNM classification in the 8th edition of the American Joint Committee on Cancer (AJCC) guidelines [8]. ABC included unresectable locally ABC and MBC.

Immunohistochemical criteria: Estrogen receptor (ER) and progesterone receptor (PR) status were assessed via immunohistochemistry. Nuclear staining ≥ 1% was defined as ER or PR positive, and HR positivity was determined either ER or PR was positive [9]. HER2 status was evaluated using the American Society of Clinical Oncology/College of American Pathologists (ASCO/CAP) guidelines [10, 11]. HER2 negativity was defined as HER2 0, 1+, or 2 + with negative fluorescence in situ hybridization (FISH). FISH negativity criteria included: HER2/CEP17 ratio<2.0 with an average HER2 copy number<4.0; or HER2/CEP17 ratio<2.0 with an average HER2 copy number ≥ 6.0. Based on the Chinese Society of Clinical Oncology (CSCO) guidelines [12], tumors were further classified as Luminal A or Luminal B (HER2-negative): Luminal A: ER-positive, PR-positive with high expression, and low Ki-67 expression. Luminal B (HER2-negative): ER-positive, PR-negative or low expression, and high Ki-67 expression.

Efficacy evaluation: Treatment response was assessed using RECIST v1.1 criteria [13]. Baseline and final imaging reports were compared, with at least one measurable lesion or osteolytic change required for evaluation during follow-up.

Definition of endocrine resistance: Primary endocrine resistance: Disease recurrence within 2 years of adjuvant ET or progression within 6 months of first-line ET for advanced disease. Acquired resistance: Recurrence between 2 years after adjuvant ET and 1 year post-treatment completion, or progression > 6 months after initiating first-line ET for advanced disease. Endocrine sensitivity: Recurrence>1 year after completing ET, including patients naive to prior ET [14].

aCCI: The Charlson Comorbidity Index (CCI) is a validated prognostic tool designed to evaluate the impact of comorbid conditions (excluding the primary disease under treatment) on 10-year survival outcomes. The aCCI is derived by incorporating age-related adjustments into the original CCI scoring framework. A higher aCCI score correlates with poorer clinical prognosis and reduced long-term survival rates, reflecting the cumulative burden of comorbidities and advancing age on patient outcomes [15].

Definition of only Lymph Node Metastasis: Only lymph node metastases included superficial (axillary, supraclavicular, infraclavicular, cervical, et al.) or deep lymph nodes (mediastinal, abdominal, et al.).

Adverse reactions (AEs) Assessment: AEs were graded and categorized per the Common Terminology Criteria for AEs (CTCAE) version5.0 [16].

### Medication regimen

The dosing regimens of CDK4/6i were selected in accordance with guideline recommendations [17]. Abemaciclib was administered at 150 mg twice daily (BID), dalpiciclib at 150 mg once daily (QD), and palbociclib at 125 mg QD, with dalpiciclib and palbociclib administered continuously for three weeks followed by a one-week treatment hiatus, thus forming a 28-day treatment cycle. Given the delayed market approval of ribociclib in China and limited national health insurance coverage, its clinical use was extremely rare. Therefore, such cases were not included in this retrospective study. Initial doses were categorized as either guideline-recommended or reduced, with clinicians selecting the appropriate regimen based on individual patient characteristics (e.g., performance status, organ function). During treatment, CDK4/6i doses were adjusted according to tolerance thresholds, including temporary interruptions or reductions to manage AEs. Per NCCN guidelines [18], resumption of therapy after interruption required confirmation of recovery based on laboratory parameters and symptomatic improvement.

### Endpoints

The primary endpoint of this study was PFS, defined as the time from initiation of CDK4/6i therapy to disease progression or all-cause death. Tumor progression was assessed by imaging of measurable lesions (e.g., CT, MRI, bone scan) every 3 months. To ensure consistency, identical imaging techniques were used for serial comparisons to confirm disease progression. Secondary endpoints included OS, ORR, and CBR. OS was calculated from initiation of CDK4/6i therapy until death or study closure. Tumor response was categorized according to RECIST v1.1 criteria into four classifications: complete response (CR), partial response (PR), stable disease (SD), and progressive disease (PD). ORR was defined as the proportion of patients achieving CR or partial response among the evaluable cohort (CR + partial response)/N, while CBR included patients with CR, partial response, or SD lasting for at least 6 months.

### Statistical analysis

PFS and OS were estimated using the Kaplan–Meier method. All missing data in the present study were handled by multiple imputation. Missing data were assumed to follow the missing-at-random (MAR) mechanism. A fully conditional specification imputation model was applied, incorporating all baseline demographic, clinical, and tumor characteristics that were potentially associated with missing variables and survival outcomes. We generated five imputed datasets with 20 iterations per imputation cycle to ensure model convergence. Pooled statistical results across the five imputed datasets were finally obtained using Rubin’s rules to integrate both within-imputation and between-imputation variability. Univariate and multivariate Cox proportional hazards regression models were performed to screen independent prognostic factors. To minimize potential confounding and selection biases, an expanded set of adjusted covariates was included in the multivariate model. Variables with significant differences in univariate analysis were subjected to collinearity diagnostics, and variables with obvious collinearity were excluded prior to multivariate regression modeling. The associations between tumor response and clinicopathological features were evaluated using the chi-square test or Fisher’s exact test as appropriate. All statistical tests were two-sided, and a *P*<0.05 was considered statistically significant. All analyses were performed using SPSS software (version 26.0).

## Results

### Patients and treatment administration

**Fig. 1:**
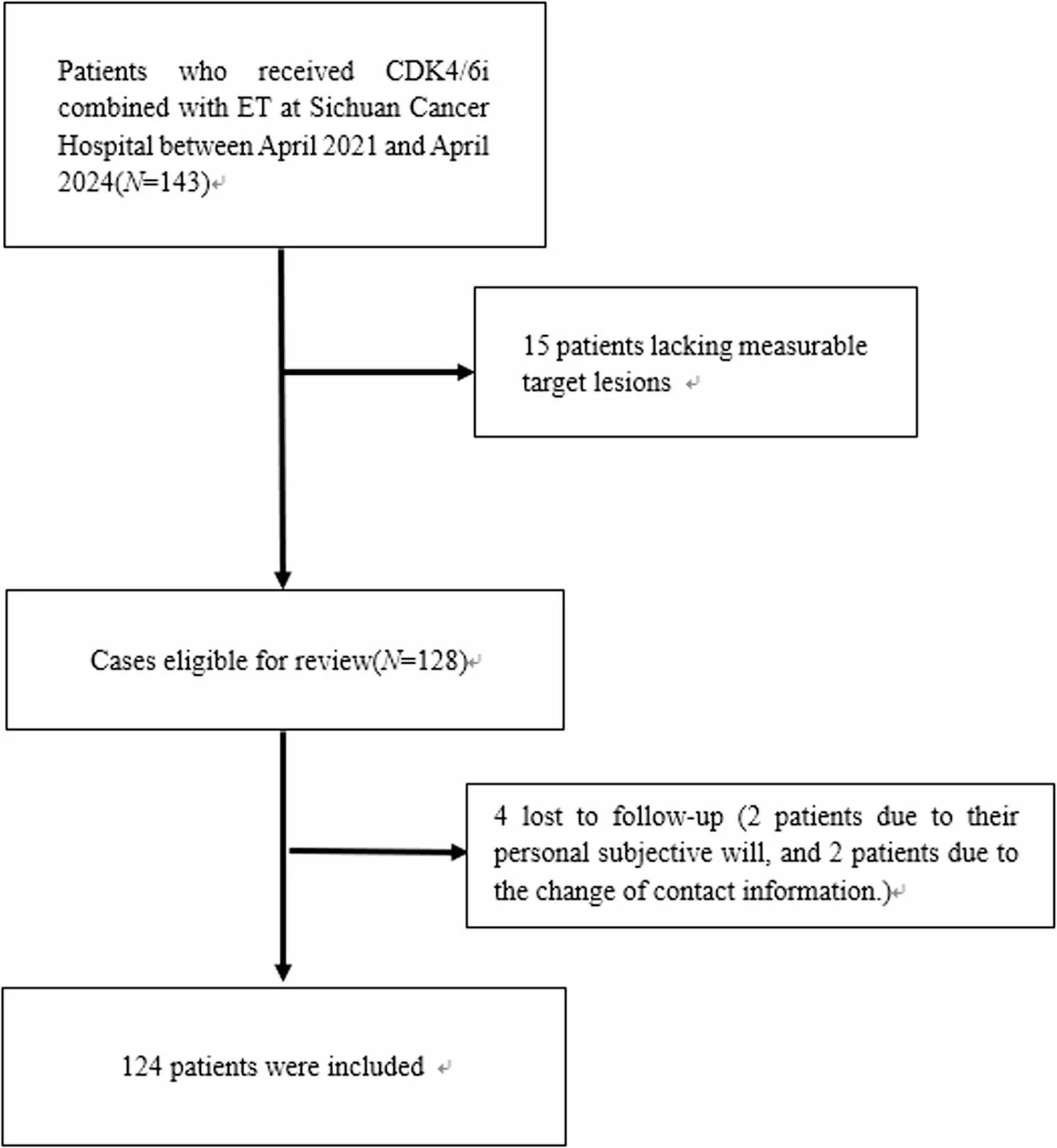
The flow chart of screening patients

A total of 143 patients with HR+/HER2- ABC receiving CDK4/6i combined with ET were identified between April 2021 and April 2024. After excluding 15 patients with no measurable target lesions and 4 patients lost to follow-up, 124 patients (including 3 males) were included in the final analysis, with a median follow-up duration of 23.0 months (95% *CI*: 16.9–29.2 months). (Fig. 1) The cohort exhibited a median age of 56 years (range: 22–84, 56.8 ± 11.4), including 10 patients (8.1%) with locally ABC and 114 (91.9%) with MBC. Missing data mainly occurred in variables of visceral metastasis status and baseline HR expression status, with an overall missing rate of 0.3% in the whole dataset. Regarding menopausal status, 32 patients (25.8%,32/124) were premenopausal and 89 (71.8%,89/124) were postmenopausal. Visceral metastases were present in 50.8% (63/124) of cases, with 93.7% (59/63) having>2 visceral metastatic sites. Only bone metastases were present in 34 patients (27.4%), while 16 (12.9%) had only lymph node/chest wall soft tissue involvement. At baseline, 8 patients (6.5%) had brain metastases, 50% of whom had received cranial radiotherapy. The type of CDK4/6i included abemaciclib (55.6%, 69/124), dalpiciclib (28.2%, 35/124), and palbociclib (16.2%, 20/124). Concomitant endocrine therapies comprised fulvestrant (54.8%, 68/124) and AIs (45.2%, 56/124). Baseline comorbidities were evaluated using the aCCI, revealing that 72.6% of patients had an aCCI<10. Regarding treatment lines, 71 patients (57.3%) received CDK4/6i-based therapy as first-line, 39 (31.5%) as second-line, and 14 (11.2%) as third-line or beyond (Table 1).

**Table 1:** Demographics and clinicopathologic characteristics

	Total *n* = 124(%)		First-line *n* = 71(%)	Second-line *n* = 39(%)	Later-lines *n* = 14(%)
Age	≤40	11(8.9)	6(8.5)	5(12.8)	0(0)
	> 40	113(91.1)	65(91.5)	34(87.2)	14(100)
Menstrual status	Premenopausal	32(25.8)	21(29.6)	11(28.2)	0(0)
	Menopausal	89(71.8)	49(69)	27(69.2)	13(92.9)
	Male	3(2.4)	1(1.4)	1(2.6)	1(7.1)
Disease stage at initial diagnosis	1	6(4.4)	3(4)	2(5.1)	1(7.1)
	2	36(29)	26(36.6)	7(17.9)	3(21.5)
	3	37(29.8)	23(32.3)	9(23.1)	5(35.7)
	4	45(36.8)	19(27.1)	21(53.9)	5(35.7)
Disease stage at current diagnosis	3	10(8.1)	9(12.7)	1(2.6)	0(0)
	4	114(91.9)	62(87.3)	38(97.4)	14(100)
Molecular typing at initial diagnosis	Luminal A	83(66.9)	47(66.2)	30(76.9)	6(42.9)
	Luminal B(HER2-)	41(33.1)	24(33.8)	9(23.1)	8(57.1)
PR	Positive	20(16.1)	11(15.5)	6(15.4)	3(21.5)
	Negative	104(83.9)	60(84.5)	33(84.6)	11(78.5)
Ki67	< 20	47(37.9)	24(33.8)	19(48.7)	4(28.6)
	≥20	77(62.1)	47(66.2)	20(51.3)	10(71.4)
HER2	0	37(29.8)	22(31)	13(33.3)	2(14.3)
	1+	35(28.2)	20(28.2)	10(25.6)	5(35.7)
	2+/FISH-	52(42)	29(40.8)	16(41.1)	7(50)
Radiotherapy	No	36(29)	21(29.6)	11(28.2)	4(28.6)
	Yes	88(71)	50(70.4)	28(71.8)	10(71.4)
Visceral metastasis	No	61(49.2)	45(63.4)	15(38.5)	1(7.1)
	Yes	63(50.8)	26(36.6)	24(61.5)	13(92.9)
Number of sites for Visceral metastasis	0	61(49.2)	45(63.4)	15(38.5)	1(7.1)
	1	52(41.9)	23(32.3)	19(48.7)	10(71.4)
	2	7(5.6)	1(1.4)	4(10.2)	2(14.4)
	≥3	4(3.3)	2(2.9)	1(2.6)	1(7.1)
Only bone metastases	No	90(72.6)	47(66.2)	31(79.5)	12(85.7)
	Yes	34(27.4)	24(33.8)	8(20.5)	2(14.3)
Bone metastases	No	43(65.3)	32(45.1)	8(20.5)	3(21.5)
	Yes	81(34.7)	39(54.9)	31(79.5)	11(78.5)
Liver metastases	No	93(75)	60(84.5)	25(64.1)	8(57.1)
	Yes	31(25)	11(15.5)	14(35.9)	6(42.9)
Brain metastases	No	116(93.5)	69(97.1)	36(92.3)	11(78.5)
	Yes	8(6.5)	2(2.9)	3(7.7)	3(21.5)
Only lymph node/chest wall soft tissue metastases	No	108(87.1)	56(78.9)	38(97.4)	14(100)
	Yes	16(12.9)	15(21.1)	1(2.6)	0(0)
Concomitant ET	Fulvestrant	68(54.8)	34(47.9)	25(64.1)	9(64.3)
	Letrozole	39(31.5)	28(39.4)	10(25.6)	1(7.1)
	Anastrozole	3(2.4)	1(1.4)	1(2.6)	1(7.1)
	Exemestane	14(11.3)	8(11.3)	3(7.7)	3(21.5)
Initial dose	recommended	110(88.7)	64(90.1)	36(92.3)	10(71.4)
	reduced	14(11.3)	7(9.9)	3(7.7)	4(28.6)
aCCI score	< 10	90(72.6)	54(76.1)	26(66.7)	10(71.4)
	≥10	34(27.4)	17(23.9)	13(33.3)	4(28.6)
Sensitivity to ET	Sensitivity and acquired resistance	92(74.2)	53(74.6)	25(64.1)	14(100)
	Primary resistance	32(25.8)	18(25.4)	14(35.9)	0(0)
RECIST1.1	CR	3(2.4)	1(1.4)	2(5.1)	0(0)
	PR	54(43.5)	39(54.9)	14(35.9)	1(7.1)
	SD	7(5.6)	1(1.4)	5(12.8)	1(7.1)
	PD	60(48.5)	30(42.3)	18(46.2)	12(85.7)
Type of CDK4/6i	Abemaciclib	69(55.6)	35(49.3)	23(59)	11(78.6)
	Dalpiciclib	35(28.2)	20(28.2)	13(33.3)	2(14.3)
	Palbociclib	20(16.2)	16(22.5)	3(7.7)	1(7.1)

### The primary endpoint

After a median follow-up of 23.0 months (95% *CI*: 16.9–29.2 months), the mPFS for the entire cohort was 20.0 months (95% *CI*: 16.0–24.0 months), and the median treatment line was first-line. A total of 60 patients experienced disease progression during the study. In the subgroup analysis stratified by treatment lines, patients who received first- or second-line CDK4/6i plus ET for ABC achieved an mPFS of 22.0 months (95% *CI*: 16.3–27.7 months), whereas those treated in later lines (≥ 3rd line) had a significantly shorter mPFS of 13.0 months (95% *CI*: 9.2–16.8 months).

**Fig. 2:**
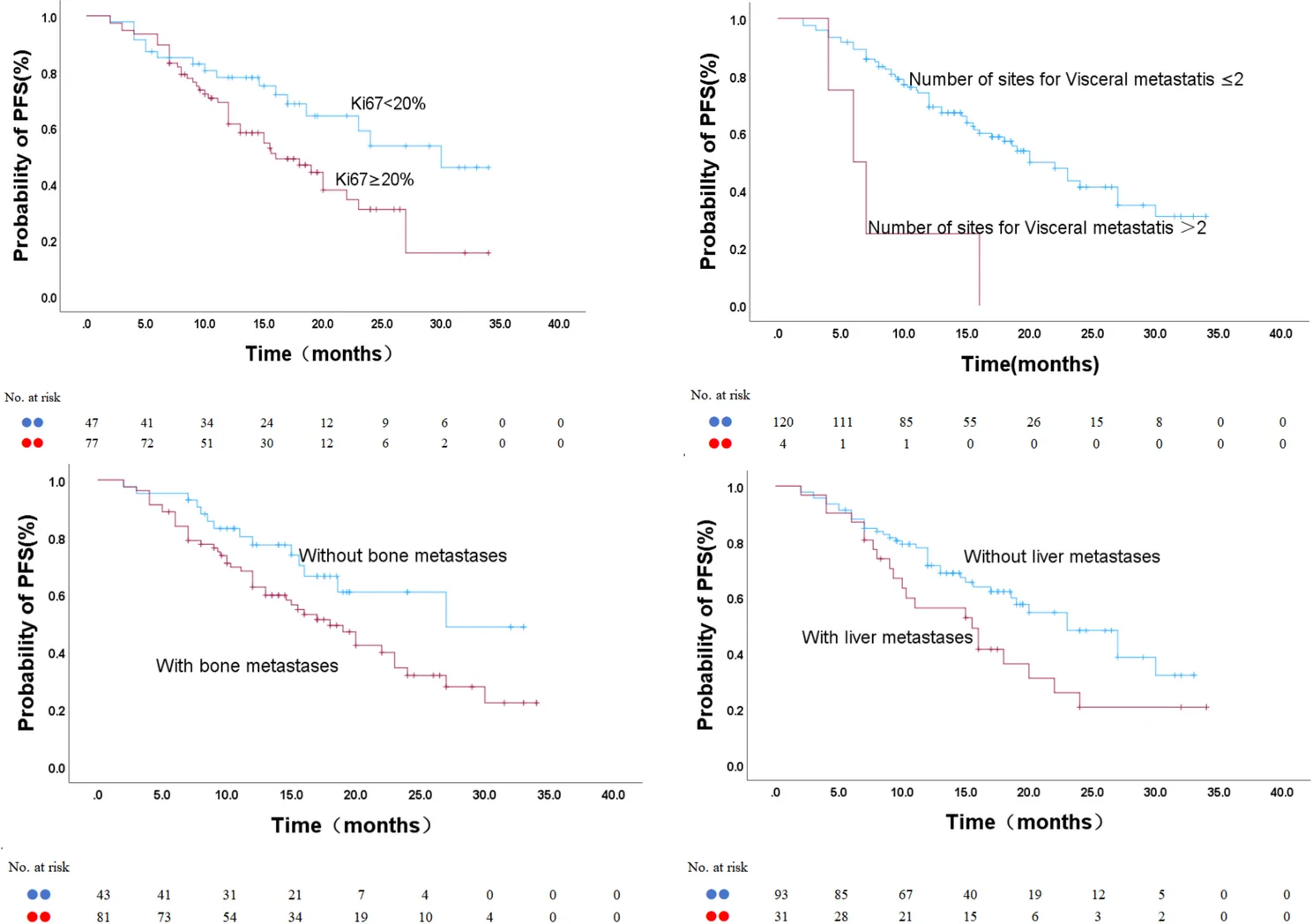
PFS of CDK4/6i plus ET stratified by different characteristics

**Fig. 3:**
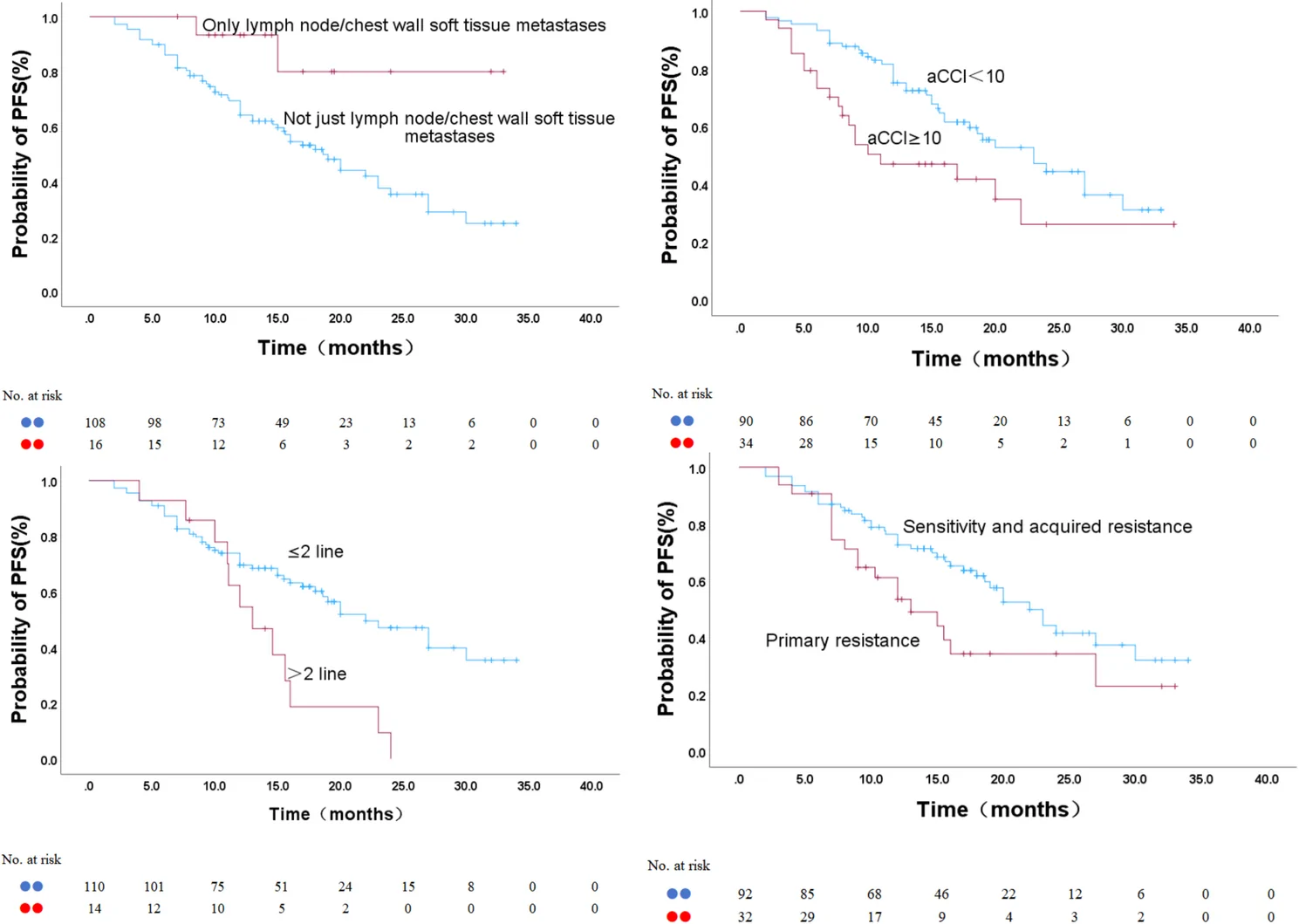
PFS of CDK4/6i plus ET stratified by different characteristics (continued)

Univariate analysis was performed to evaluate the impact of clinicopathological factors on PFS. The results demonstrated that Ki-67 expression, number of visceral metastases, presence of liver metastases, bone metastases, only lymph node or chest wall metastases, line of CDK4/6i therapy, aCCI score, and sensitivity to ET were all statistically significant predictors of PFS (*P*<0.05). Specifically, patients with Ki-67 <20%, ≤ 2 visceral metastatic sites, only lymph node or chest wall metastases, absence of liver metastases, absence of bone metastases, ≤ 2 prior lines of therapy, aCCI<10, and endocrine-sensitive or acquired endocrine-resistant disease had significantly greater survival benefits (Table 2; Figs. 2 and 3).

**Table 2:** Univariate analyses of factors predicting PFS in patients treated with CDK4/6i

Characteristic		*N*	mPFS	95% CI	χ²	*P*
Age	≤40	11	27	17.1–36.9	0.846	0.358
	> 40	113	20	14.6–25.4		
Menstrual status	Premenopausal	32	19	9.9–28.1	0.105	0.949
	Menopausal	89	20	14.3–25.7		
	Male	3	11	*NR*		
Molecular typing at initial diagnosis	Luminal A	83	22	17.5–26.5	2.148	0.143
	Luminal B(HER2-)	41	16	12.4–19.6		
PR	Positive	20	20	13.9–26.1	0.001	0.98
	Negative	104	20	15.5–24.5		
Ki67	< 20	47	30	*NR*	5.604	0.018
	≥20	77	16	10.8–21.2		
HER2	0	37	20	11.4–28.6	0.711	0.399
	Else	87	20	14.1–25.9		
Radiotherapy	No	36	23	6.44–39.6	0.111	0.739
	Yes	88	20	14.3–25.7		
Visceral metastasis	No	61	30	15.1–44.9	0.395	0.238
	Yes	63	18	14.2–21.8		
Number of sites for Visceral metastasis	≤2	120	20	16.0–24.0	10.82	0.001
	> 2	4	6	3.1–8.9		
Only bone metastases	No	90	20	14.9–25.1	0.185	0.667
	Yes	34	19	7.8–30.2		
Bone metastases	No	43	27	*NR*	4.282	0.039
	Yes	81	18	13.8–22.2		
Liver metastases	No	93	23	17.2–28.8	4.178	0.041
	Yes	31	15.5	9.3–21.7		
Brain metastases	No	116	20	16.0–24.0	2.781	0.095
	Yes	8	11	2.7–19.3		
Only lymph node/chest wall soft tissue metastases	No	108	18.6	15.0-22.2	5.777	0.016
	Yes	16	*NR*	*NR*		
Line of CDK4/6i	≤2	110	22	16.3–27.7	7.542	0.006
	> 2	14	13	9.2–16.8		
Concomitant ET	Fulvestrant	68	18	13.7–22.3	2.564	0.109
	AI	56	*NR*	*NR*		
Initial dose	recommended	110	20	16.3–23.7	0.077	0.781
	reduced	14	15.6	0-33.2		
aCCI score	< 10	90	23	16.9–29.1	6.026	0.014
	≥10	34	11	3.0–19.0		
Sensitivity to ET	Sensitivity and acquired resistance	92	22	18.1–25.9	3.945	0.047
	Primary resistance	32	13	7.9–18.1		
Type of CDK4/6i	Abemaciclib	69	18.6	14.3–22.9	2.407	0.300
	Dalpiciclib	35	22	*NR*		
	Palbociclib	20	*NR*	*NR*		

Collinearity diagnostics were performed for the clinicopathological factors identified as statistically significant in the univariate analysis, and no significant collinearity was detected among these variables. Subsequently, all factors were incorporated into a multivariate Cox regression analysis. The results revealed that Ki-67, line of CDK4/6i, aCCI score, and sensitivity to ET emerged as independent prognostic factors for PFS (*P*<0.05) in the present study (Table 3).

**Table 3:** Multivariate analyses of factors predicting PFS in patients treated with CDK4/6i

Characteristic	Wald	HR	95% CI	*P*
Ki67	4.208	1.828	1.027–3.252	0.04
Number of sites for Visceral metastasis	3.214	3.067	0.901–10.441	0.073
Bone metastases	1.339	1.494	0.757–2.947	0.247
Liver metastases	0.258	0.844	0.438–1.624	0.612
Only lymph node/chest wall soft tissue metastases	1.458	0.396	0.088–1.782	0.227
Line of CDK4/6i	5.789	2.487	1.184–5.225	0.016
aCCI score	7.327	2.367	1.268–4.418	0.007
Sensitivity to ET	7.215	2.405	1.268–4.561	0.007

### The secondary endpoint

**Fig. 4:**
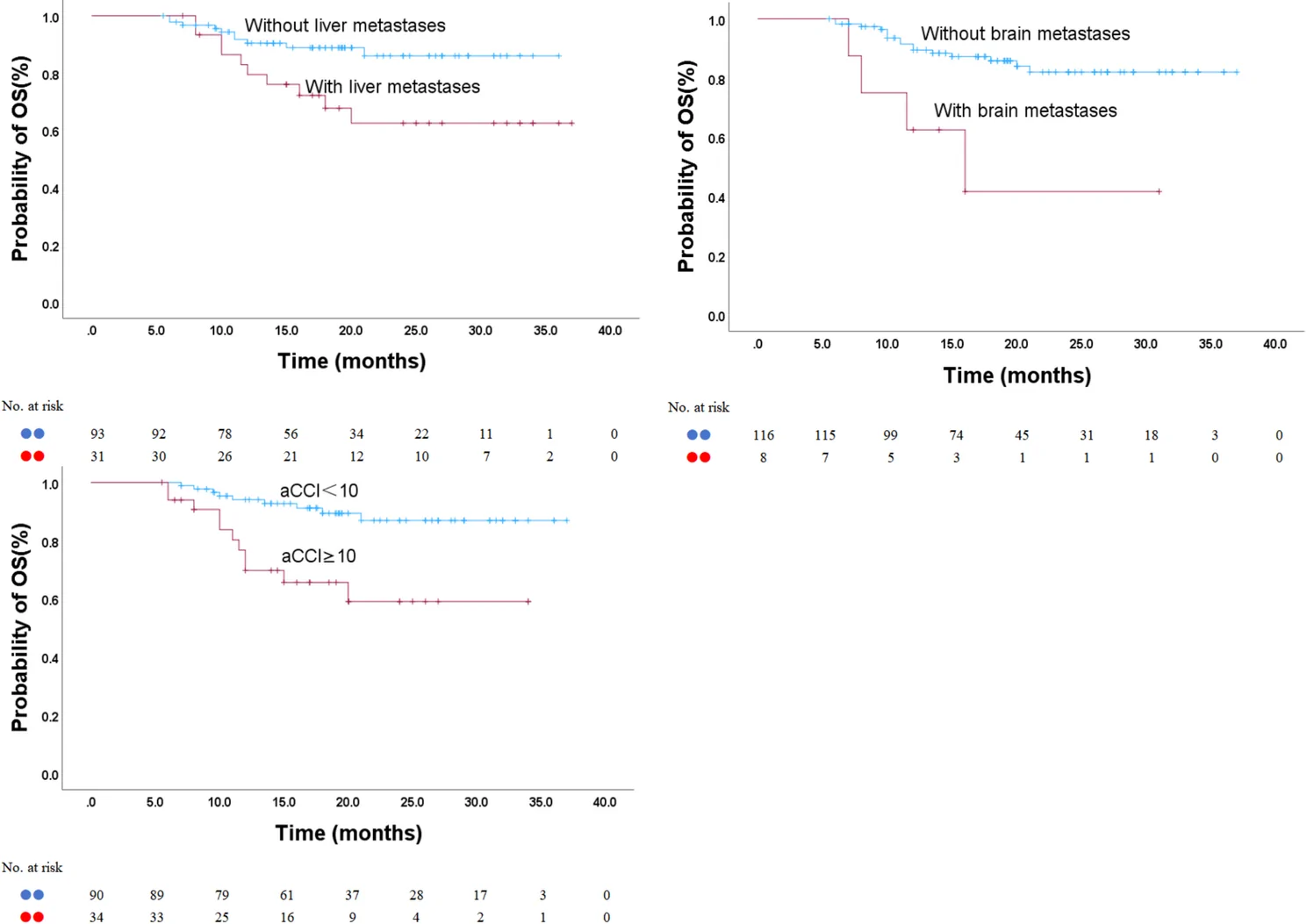
OS of CDK4/6i plus ET stratified by different characteristics

At the last follow-up in October 2024, 20 patients (16.1%) had died. Due to the relatively short follow-up period, the mOS for the entire cohort was not reached. Among the entire cohort, only patients with brain metastases had a measurable mOS of 16.0 months (95% *CI*: 7.3–24.7 months). Univariate analysis showed that patients without liver metastases, absence of brain metastases, and aCCI<10 derived significantly greater OS outcomes (*P*<0.05). Subsequent multivariate Cox regression analysis incorporating these statistically significant variables identified aCCI score as an independent predictor of OS in our cohort (Tables 4 and 5; Fig. 4).

**Table 4:** Univariate analyses of factors predicting OS in patients treated with CDK4/6i

Characteristic		*N*	mOS	95% CI	χ²	*P*
Age	≤40	11	*NR*	*NR*	2.433	0.119
	> 40	113	*NR*	*NR*		
Menstrual status	Premenopausal	32	*NR*	*NR*	1.974	0.373
	Menopausal	89	*NR*	*NR*		
	Male	3	11.5	*NR*		
Molecular typing at initial diagnosis	Luminal A	83	*NR*	*NR*	0.066	0.797
	Luminal B(HER2-)	41	*NR*	*NR*		
PR	Positive	20	*NR*	*NR*	0.063	0.802
	Negative	104	*NR*	*NR*		
Ki67	< 20	47	*NR*	*NR*	2.55	0.11
	≥20	77	*NR*	*NR*		
HER2	0	37	*NR*	*NR*	0.798	0.372
	else	87	*NR*	*NR*		
Radiotherapy	No	36	*NR*	*NR*	1.653	0.199
	Yes	88	*NR*	*NR*		
Visceral metastasis	No	61	*NR*	*NR*	2.582	0.108
	Yes	63	*NR*	*NR*		
Number of sites for Visceral metastasis	≤2	120	*NR*	*NR*	3.165	0.075
	> 2	4	16	*NR*		
Only bone metastases	No	90	*NR*	*NR*	0.06	0.806
	Yes	34	*NR*	*NR*		
Bone metastases	No	43	*NR*	*NR*	0.187	0.666
	Yes	81	*NR*	*NR*		
Liver metastases	No	93	*NR*	*NR*	3.112	0.013
	Yes	31	*NR*	*NR*		
Brain metastases	No	116	*NR*	*NR*	9.357	0.002
	Yes	8	16	7.3–24.7		
Only lymph node/chest wall soft tissue metastases	No	108	*NR*	*NR*	2.799	0.094
	Yes	16	*NR*	*NR*		
Line of CDK4/6i	≤2	110	*NR*	*NR*	0.977	0.323
	> 2	14	*NR*	*NR*		
Concomitant ET	Fulvestrant	68	*NR*	*NR*	2.103	0.147
	AI	56	*NR*	*NR*		
Initial dose	recommended	110	*NR*	*NR*	0.263	0.608
	reduced	14	*NR*	*NR*		
aCCI score	< 10	90	*NR*	*NR*	11.453	0.001
	≥10	34	*NR*	*NR*		
Sensitivity to ET	Sensitivity and acquired resistance	92	*NR*	*NR*	3.025	0.082
	Primary resistance	32	*NR*	*NR*		
Type of CDK4/6i	Abemaciclib	69	*NR*	*NR*	0.017	0.992
	Dalpiciclib	35	*NR*	*NR*		
	Palbociclib	20	*NR*	*NR*		

**Table 5:** Multivariate analyses of factors predicting OS in patients treated with CDK4/6i

Characteristic	Wald	HR	95% CI	*P*
Liver metastases	0.740	1.539	0.576–4.110	0.390
aCCI score	5.509	3.140	1.208–8.164	0.019
Brain metastases	3.600	3.096	0.963–9.948	0.058

Based on RECIST v1.1 criteria, bone metastases were classified as non-measurable or non-evaluable for therapeutic response. Therefore, patients with only bone metastases were excluded when analyzing correlations between clinicopathological factors and tumor response, resulting in 90 evaluable patients. Among these, 3 patients (3.3%) achieved CR, 39 (43.3%) partial response, 6 (6.7%) SD, and 42 (46.7%) PD. The ORR was 46.7%, and the CBR was 53.3%. In patients treated with first- or second-line therapy, ORR and CBR were 52.6% and 59.0%, respectively, versus 8.3% and 16.7% in those who received later-line therapy. Further analysis of correlations between clinicopathological factors and tumor response showed that visceral metastases (present/absent), bone/liver metastases, only lymph node or chest wall metastases, CDK4/6i treatment line, and CDK4/6i type were significantly associated with both ORR and CBR (*P*<0.05). Additionally, the number of visceral metastatic sites correlated solely with CBR, while the specific ET combined with CDK4/6i and sensitivity to ET were exclusively associated with ORR (Table 6).

**Table 6:** Analysis of the association between factors and ORR/CBR

Characteristic		ORR			CBR		
		*N*(%)	χ2	*P*	*N*(%)	χ2	*P*
Age			0.79	0.62		0.422	0.516
	≤40	1(25)			1(25)		
	> 40	41(47.7)			47(54.7)		
Menstrual status			1.131	0.557		0.877	0.645
	Premenopausal	6(37.5)			7(43.8)		
	Menopausal	34(47.9)			39(54.9)		
	Male	2(66.7)			2(66.7)		
Molecular typing at initial diagnosis			1.203	0.273		0.465	0.495
	Luminal A	30(50.8)			33(55.9)		
	Luminal B(HER2-)	12(38.7)			15(48.4)		
PR			0.254	0.614		0.001	0.971
	Positive	35(47.9)			9(52.9)		
	Negative	7(41.2)			39(53.4)		
Ki67			2.491	0.114		3.722	0.054
	< 20	19(57.6)			22(66.7)		
	≥20	23(40.4)			26(45.6)		
HER2			0.376	0.539		0.705	0.401
	0	12(52.2)			14(60.9)		
	Else	30(44.8)			34(50.7)		
Radiotherapy			0.757	0.384		0.004	0.95
	No	14(53.8)			14(53.8)		
	Yes	28(43.8)			34(53.1)		
Visceral metastasis			11.396	0.001		6.259	0.012
	No	21(72.4)			21(72.4)		
	Yes	21(34.4)			27(44.3)		
Number of sites for Visceral metastasis			3.663	0.056		4.784	0.029
	≤2	42(48.8)			48(55.8)		
	> 2	0(0)			0(0)		
Bone metastases			8.601	0.003		6.585	0.01
	No	27(62.8)			29(67.4)		
	Yes	15(31.9)			19(40.4)		
Liver metastases			8.268	0.004		8.439	0.004
	No	34(57.6)			38(64.4)		
	Yes	8(25.8)			10(32.3)		
Brain metastases			2.749	0.097		0.324	0.569
	No	41(50.0)			45(54.9)		
	Yes	1(12.5)			3(37.5)		
Only lymph node/chest wall soft tissue metastases			13.036	<0.001		9.127	0.003
	No	28(37.8)			34(45.9)		
	Yes	14(87.5)			14(87.5)		
Line of CDK4/6i			8.175	0.004		7.479	0.006
	≤2	41(52.6)			46(59.0)		
	> 2	1(8.3)			2(16.7)		
Concomitant ET			4.464	0.035		2.857	0.091
	Fulvestrant	16(35.6)			20(44.4)		
	AI	26(57.8)			28(62.2)		
Initial dose			0.534	0.465		1.45	0.229
	recommended	38(48.1)			44(55.7)		
	reduced	4(36.3)			4(36.4)		
aCCI score			1.437	0.231		0.417	0.519
	< 10	32(50.8)			35(55.6)		
	≥10	10(37.0)			13(48.1)		
Sensitivity to ET			4.027	0.045		3.296	0.069
	Sensitivity and acquired resistance	35(53.0)			39(59.1)		
	Primary resistance	7(29.2)			9(37.5)		
Type of CDK4/6i			12.573	0.002		10.778	0.005
	Abemaciclib	15(30.0)			19(38.0)		
	Dalpiciclib	16(66.7)			18(75.0)		
	Palbociclib	11(68.8)			11(68.8)		

### AEs

AEs during CDK4/6i combination therapy were graded per CTCAE v5.0. The most common hematological toxicities were leukopenia (83.1%) and neutropenia (81.5%), which were predominantly grade 1–2. Non-hematological toxicities differed by CDK4/6i agent. Stratified by CDK4/6i agent, diarrhea was the most common AE with abemaciclib (78.3%), fatigue or alopecia with dalpiciclib (48.6%), and nausea or anorexia with palbociclib (57.9%). Treatment interruption due to AEs occurred in 34 patients (27.4%), including 8 (6.5%) who discontinued permanently or switched treatments and 3 (2.4%) requiring dose reduction (Table 7). Dalpiciclib is a novel CDK4/6i developed in China with limited international real-world evidence for its use. In our study, among neutropenia events, 12 patients developed grade ≥ 3, accounting for 12/29 (41.4%). Additionally, 7 patients (21.9%, 7/32) had grade ≥ 3 leukopenia. Although the incidence of grade ≥ 3 neutropenia and leukopenia of dalpiciclib was relatively higher than that of other CDK4/6is, no new safety signals were identified, and these toxicities are clinically manageable hematological adverse events consistent with the overall trend of the same class of drugs.

**Table 7:** AEs of all grades of CDK4/6i-based regimens

		All grades(%)	Grade1 ~ 2(%)	Grade3(%)	Grade4(%)
Hematologic					
	Anemia	45(36.3)	38(30.6)	6(4.8)	1(0.8)
	Leukopenia	103(83.1)	94(75.8)	17(13.7)	2(1.6)
	Neutropenia	101(81.5)	74(59.7)	24(19.4)	3(2.4)
	Thrombocytopenia	36(29.0)	32(25.8)	1(0.8)	3(2.4)
Nonhematologic					
	Nausea	33(26.6)	33(26.6)	0(0)	0(0)
	vomit	6(4.8)	6(4.8)	0(0)	0(0)
	Diarrhea	60(48.4)	54(43.5)	6(4.8)	0(0)
	Alopecia	33(26.6)	33(26.6)	0(0)	0(0)
	Fatigue	47(37.9)	47(37.9)	0(0)	0(0)
	Numbness	14(11.3)	14(11.3)	0(0)	0(0)
	Rash	2(1.6)	2(1.6)	0(0)	0(0)
	Palpitation	3(2.4)	3(2.4)	0(0)	0(0)
	Oral mucositis	2(1.6)	2(1.6)	0(0)	0(0)
	Constipation	2(1.6)	2(1.6)	0(0)	0(0)
	Arthralgia	8(6.5)	8(6.5)	0(0)	0(0)
	Pruritus	2(1.6)	2(1.6)	0(0)	0(0)
	Creatinine increased	72(58.1)	72(58.1)	0(0)	0(0)
	Thrombotic events	1(0.8)	1(0.8)	0(0)	0(0)
	ALT increased	45(36.3)	44(35.5)	1(0.8)	0(0)

The aCCI assesses comorbidity burden and mortality risk, with higher scores indicating poorer clinical status, potentially predisposing patients to increased treatment-related toxicities [15]. To evaluate the association between aCCI score and treatment discontinuation, a Chi-square test was performed. Among patients with aCCI<10, 21 (23.3%) had treatment interruptions, compared to 13 (38.2%) in the aCCI ≥ 10 group, though this difference was not statistically significant (*P* = 0.097).

## Discussion

In the pre-CDK4/6i era, HR+/HER2- ABC management relied primarily on chemotherapy and ET, with suboptimal outcomes largely due to endocrine resistance—a major therapeutic challenge [19]. Studies such as the FIRST [2], FALCON [3], and FRIEND [20] trials demonstrated that fulvestrant improved PFS compared to traditional endocrine regimens. Since 2015, the introduction of CDK4/6is into clinical practice has ushered in the era of ET combined with targeted therapy for HR+/HER2- ABC. The SONABRE study [21] reported a shift in RWD treatment patterns between 2008 and 2019: ET and targeted therapy utilization increased, while chemotherapy declined, with CDK4/6i utilization rising from 0% (2008–2010) to 54% (2017–2019). Concurrently, mOS improved from 31.1 months (2008–2010) to 38.4 months (2017–2019), highlighting the transformative survival benefits of CDK4/6i in this population.

While pivotal clinical trials have established the efficacy of CDK4/6i-ET combination in HR+/HER2- ABC, their strict inclusion/exclusion criteria limit generalizability to RWD settings, necessitating further exploration of efficacy and safety in clinical practice [22]. This retrospective study evaluated RWD outcomes of CDK4/6i-ET in HR+/HER2- ABC. Our findings differ from those of previous RWD reports. Müller et al. [23] reported an mPFS of 17 months in a German cohort (62.1% in first line), while Binliang Liu [24] observed a shorter mPFS of 12.8 months (26.7% in first line). These outcomes were slightly lower than those observed in our cohort. Further analysis revealed that the two aforementioned studies enrolled populations with a higher proportion of visceral metastases (63.2% and 54.7%, respectively), both higher than the 50.8% rate in our cohort. The secondary endpoint of mOS was not reached, likely due to insufficient follow-up. Similarly, the Chinese multicenter YOUNGBC-28 study [25] reported an mPFS of 19 months (95% *CI*: 15.0–22.5) for first-line CDK4/6i-ET, with no significant OS difference across different CDK4/6is (*P* = 0.84). Contrasting with RCTs, RWD studies such as P-REALITY [26] and P-REALITY X [27] demonstrated OS benefits with palbociclib-ET, potentially attributable to longer follow-up and higher rates of subsequent CDK4/6i rechallenge in later-line therapy, which may amplify cumulative OS gains. The visceral metastasis rate in our cohort was 50.8%, marginally lower than the 63.2% and 54.7% reported in previous studies including YOUNGBC‑28. Such discrepancy was mainly attributed to differences in study population selection and baseline characteristics: the YOUNGBC‑28 trial enrolled younger patients with a higher proportion of de novo metastasis and more aggressive tumor phenotypes, whereas our cohort covered all age groups with a median age of 56 years and a relatively lower overall disease risk, and also included more patients with bone-only metastases or well-controlled systemic conditions at enrollment. As a single-center study conducted in economically underdeveloped western China, disparities in regional economic levels, early self-payment policy for CDK4/6is, and inconsistent physicians’ judgment on tumor aggressiveness and visceral crisis also led some visceral metastasis patients to receive upfront chemotherapy for rapid symptom relief, further affecting the enrolled proportion of visceral metastatic cases. The relatively lower visceral tumor burden in our study may partially explain the favorable mPFS of 20.0 months, since visceral metastasis especially liver metastasis predicts rapid disease progression and poor prognosis, while patients with predominant bone metastasis usually exhibit indolent progression and better treatment responses. These inherent differences in visceral metastasis distribution may introduce heterogeneity to study outcomes; thus, our findings should be interpreted with caution, and further multicenter, prospective, large-sample real-world studies are warranted to validate and generalize our results alongside nationwide insurance coverage of CDK4/6is in China.

Among the 124 patients included in this study, 69 (55.6%) received abemaciclib, likely attributable to its early inclusion in China’s national reimbursement drug list (NRDL). No significant difference in PFS was observed across CDK4/6i types (*P* = 0.300), consistent with prior studies [24, 25] and guideline recommendations [28], albeit in the absence of head-to-head randomized controlled trials (RCTs). Owing to the relatively small sample size, the statistical power to detect small differences among treatment groups may be limited. All results are reported with 95% confidence intervals to indicate the precision of effect estimates. Due to factors such as health insurance policies and drug approval timelines, the real-world data we collected for dalpiciclib and palbociclib were more limited, and we were unable to obtain relevant data for ribociclib at all. This indeed results in reduced statistical power when comparing the efficacy of different CDK4/6is. Similarly, no PFS difference was detected between fulvestrant and AI as the endocrine partner (*P* = 0.109), aligning with findings from the PARSIFAL trial [29], which reported comparable efficacy for palbociclib combined with fulvestrant versus letrozole in endocrine-sensitive HR+/HER2- ABC. Current guidelines [30] recommend CDK4/6i combined with AI as first-line therapy for AI-naive patients and CDK4/6i plus fulvestrant following AI failure. Intriguingly, RECIST 1.1-based tumor response assessments revealed inferior outcomes with fulvestrant-based combinations, potentially due to its preferential use in third- or later-line therapy (64.3% vs. 35.7% in earlier lines).

Regarding prognostic factors, multivariate analysis identified Ki-67, aCCI, line of CDK4/6i, and sensitivity to ET as independent predictors of PFS, while aCCI alone independently predicted OS.

Ki-67, a nuclear protein associated with cell proliferation, quantifies the percentage of cells in the active cell cycle and serves as a critical biomarker for tumor proliferation in clinical practice [31]. Prior studies indicate that high Ki-67 expression (≥ 20%) correlates with poor prognosis [32].We found that inferior PFS in patients with Ki-67 ≥ 20% compared to those with Ki-67<20%, consistent with Muller et al. [23]. Notably, CDK4/6i conferred survival benefits regardless of Ki-67 expression levels [33]. Furthermore, neoadjuvant therapy-related studies [34, 35] suggest that persistently high Ki-67 post-treatment predicts unfavorable survival outcomes.

The CCI, introduced in 1987 to assess comorbid disease burden [15], was later aCCI in 1994 to enhance mortality risk stratification [36]. Accumulating evidence supports the prognostic value of aCCI in oncology [37–40]. Our study identified aCCI score as an independent predictor of both PFS and OS, with patients scoring ≥ 10 exhibiting reduced survival benefits, likely due to higher comorbidity burdens and compromised physiological reserve. Similarly, a study evaluating prognostic factors in elderly breast cancer patients [41] reported aCCI as an independent predictor of OS and non-breast cancer-specific survival (NBCSS). El Badri et al. [42] further demonstrated in a RWD analysis that aCCI>10 significantly predicted shorter PFS in ABC. Intriguingly, 50% of patients requiring initial CDK4/6i dose reduction had aCCI ≥ 10, compared to 24.5% in those receiving standard doses. This aligns with findings by El Badri et al. [42], who linked higher aCCI scores to increased incidence and severity of neutropenia, potentially attributable to impaired drug metabolism and clearance in comorbid populations. In clinical practice, for this subset of patients, reducing the initial dosage or selecting a more suitable CDK4/6i based on the patient’s specific circumstances may effectively alleviate adverse reactions during treatment, thereby reducing the rate of treatment discontinuation and ensuring therapeutic efficacy. Given the elevated comorbidity burden in elderly patients, aCCI may serve as a vital tool for pretreatment risk assessment [43, 44]. While our study, defining elderly patients as ≥ 70 years according to Chinese geriatric breast cancer guidelines [45], found no significant PFS/OS differences between elderly and younger cohorts, this null result may reflect limited statistical power due to small sample size.

Our study demonstrated superior PFS in patients receiving first- or second-line CDK4/6i therapy compared to those treated in third- or later-line settings (22.0 vs. 13.0 months; *P*<0.05), with PFS progressively declining as the line of therapy advanced. This aligns with prior studies [24, 46, 47] and may reflect preserved drug sensitivity, better tolerability, and lower tumor burden in earlier treatment lines.

Sensitivity to ET remains a critical determinant of therapeutic efficacy in HR + ABC [25]. Consistent with existing evidence, our results showed significantly prolonged mPFS in endocrine-sensitive or acquired-resistant patients versus those with primary resistance (22.0 vs. 13.0 months). The PALOMA-3 trial [5] similarly reported greater palbociclib benefit in acquired-resistant populations. Further supporting this, the TREnd trial [48] and PALBOCOMP study [49] observed enhanced survival benefits in patients with>6 months of prior ET. RWD analyses [50, 51] corroborated these findings, demonstrating longer PFS in endocrine-sensitive/ acquired-resistant cohorts (14.0 vs. 8.0 months; not reached vs. 5.9 months). Collectively, these results suggest that CDK4/6i may partially reverse endocrine resistance [52], offering clinical utility even in pretreated populations.

Survival analysis revealed that patients with brain metastases (*n* = 8) had a significantly shorter mOS of 16.0 months (95% *CI*: 7.3–24.7; *P* = 0.002) compared to those without, though multivariate analysis did not identify brain metastasis as an independent prognostic factor for OS. The blood-brain barrier (BBB), a critical interface separating the central nervous system (CNS) from systemic circulation, not only protects the CNS from pathogens but also limits drug penetration, resulting in subtherapeutic drug concentrations and diminished efficacy. Although HR+/HER2- breast cancer exhibits a lower incidence of brain metastasis than HER2 + or triple-negative breast cancer (TNBC), therapeutic strategies for established brain metastases warrant attention. Preclinical models [53] suggest superior CNS penetration of abemaciclib over other CDK4/6i. However, the JPBO trial [54] evaluating abemaciclib monotherapy in brain metastases demonstrated a disappointing intracranial ORR of 5.6%, which may partially account for the limited survival benefit in this population. Despite the limited sample size, the efficacy/safety trends observed in this subgroup were consistent with those in the overall population, providing preliminary evidence for future multicenter, large-sample studies focusing on patients with brain metastases.

Treatment-related AEs observed in this study predominantly included leukopenia (83.1%) and neutropenia (81.5%). Most toxicities were grade 1–2 in severity, with no treatment-related serious adverse events (SAEs) reported. Grade 4 leukopenia occurred in 2 patients (1.6%) and grade 4 neutropenia in 3 patients (2.4%). This safety profile aligns with previous reports from both randomized controlled trials (RCTs) and RWD studies [23, 24]. Beyond class-effect myelosuppression common to all CDK4/6i, agent-specific toxicities require vigilant monitoring. For instance, abemaciclib-associated diarrhea necessitates proactive management during treatment initiation. Apart from the above common adverse events, we also observed asymptomatic reactions such as elevated blood lipids and increased transaminase levels. These abnormalities are associated with both CDK4/6i and the combined endocrine agents [55]. Additionally, studies have found that patients with advanced disease stages tend to experience more severe adverse events after systemic therapy [56]. This phenomenon appears to be related to the substantial weight assigned to disease stage within the overall score of the aCCI.

Besides promising efficacy in ABC, CDK4/6i can also further reduce the risks of local recurrence and distant metastasis when used for adjuvant intensified therapy in appropriately selected patients with early-stage breast cancer [57]. And during the study period, constrained by multiple real-world factors such as limited drug accessibility and economic burden, chemotherapy remained the main subsequent treatment for patients with disease progression after CDK4/6i therapy. However, Given the complex molecular mechanisms of tumor initiation and progression, and with growing evidence from clinical trials plus ongoing updates to international guidelines, biomarker testing for the PAM signaling pathway, PTEN deficiency, ESR1 mutation and HER2 status has gradually offered more individualized alternative treatment options for patients with disease progression after CDK4/6i therapy [58].

This study has several limitations that warrant acknowledgment. First, given the immaturity of OS data at this stage, conclusions regarding OS still require further validation in subsequent studies. Second, there was obvious heterogeneity in treatment lines, CDK4/6i subtypes, and endocrine combination regimens. Due to the relatively small sample size, stratified subgroup analysis was not performed, because splitting the cohort would lead to underpowered statistical comparison. This issue has been acknowledged as a limitation, and larger-scale studies are required in the future to address this heterogeneity via stratified analysis. In addition, sensitivity analysis such as complete-case analysis was not performed, because excluding cases with missing data would markedly reduce the sample size and compromise statistical power. Thus, the robustness of our findings under different missing data assumptions remains unconfirmed, and larger cohorts are required for further validation. Another methodological limitation is that we excluded patients with non-measurable lesions, which may have overestimated response endpoints including ORR and CBR, as this subgroup’s distinct baseline and response features were not captured in the current analysis. This study was a retrospective single-center investigation, which is susceptible to selection bias and confounding bias. Limited to a single institutional sample and retrospective data collection, some unmeasured confounders could not be fully controlled. Due to the limited number of endpoint events in the present cohort, the events per variable (EPV) ratio of the multivariate Cox regression model was relatively low, which may introduce a potential risk of model overfitting. Therefore, the prognostic factors identified in this study should be interpreted with extreme caution. These findings are strictly exploratory and cannot be regarded as definitive clinical conclusions. Further validation based on larger-scale and independent external cohorts is required to confirm the stability and generalizability of our results.

## Conclusion

This RWD study systematically analyzed the clinical application of CDK4/6i combined with ET in HR+/HER2- ABC within the Chinese population. By incorporating diverse CDK4/6i-based regimens, our design better reflects RWD clinical prescribing patterns compared to protocol-restricted RCTs. The observed mPFS of 20.0 months aligns with landmark RCT findings. Multivariate analysis identified four independent predictors of prolonged PFS: Ki-67<20%, aCCI<10, CDK4/6i in 1st/2nd-line settings, endocrine sensitivity or acquired resistance. Notably, patients with aCCI ≥ 10 exhibited inferior long-term survival outcomes, providing actionable criteria for optimizing patient selection. The main novelty of the present study lies in the incorporation of the aCCI to quantify comorbidity burden and its prognostic impact on survival outcomes. While efficacy outcomes were consistent with previous reports, our findings highlight that comorbidity assessment using aCCI can help identify patients at higher risk of progression or death, which may facilitate risk stratification and individualized treatment selection. These results underscore the importance of routine comorbidity evaluation in clinical practice beyond traditional prognostic factors. However, due to the limited number of OS events, these findings should be interpreted with caution. And these findings warrant further validation in prospective studies. The safety profile mirrored previous reports, with leukopenia and neutropenia being predominant grade 1–2 toxicities, no treatment-related mortality occurred. Of note, this study was designed as an observational retrospective analysis. Therefore, the relevant findings regarding precision-based strategies and patient selection should be considered exploratory. The prognostic implication of aCCI needs to be validated in larger prospective cohorts, and clinical application should be performed with caution.

## Data Availability

The datasets used and/or analysed during the current study are available from the corresponding author on reasonable request.

## Abbreviations

CDK4/6i: Cyclin dependent kinase 4/6 inhibitor
HR: Hormone receptor
HER2: Human epidermal growth factor receptor 2
BC: Breast cancer
ABC: Advanced breast cancer
PFS: Progression-free survival
OS: Overall survival
ORR: Objective response rate
CBR: Clinical benefit rate
CI: Confidence interval
aCCI: Age-adjusted Charlson comorbidity index
AI: Aromatase inhibitor
RCT: Randomized Controlled Trail
RWD: Real-world
ER: Estrogen receptor
PR: Progesterone receptor
CR: Complete response
SD: Stable disease
PD: Progressive disease
NR: Not Reached
NBCSS: Non-BC-specific survival
DCR: Disease control rate
T-dxd: Trastuzumab deruxtecan
